# Fine mapping of genes within the *IDDM8 *region in rheumatoid arthritis

**DOI:** 10.1186/ar2037

**Published:** 2006-08-31

**Authors:** Anne Hinks, Anne Barton, Sally John, Neil Shephard, Jane Worthington

**Affiliations:** 1 Arthritis Research Campaign Epidemiology Unit, University of Manchester, Manchester M13 9PT, UK

## Abstract

The *IDDM8 *region on chromosome 6q27, first identified as a susceptibility locus for type 1 diabetes, has previously been linked and associated with rheumatoid arthritis (RA). The region contains a number of potential candidate genes, including programmed cell death 2 (*PDCD2*), the proteosome subunit beta type 1 (*PSMB1*), delta-like ligand 1 (*DLL-1*) and TATA box-binding protein (*TBP*) amongst others. The aim of this study was to fine map the *IDDM8 *region on chromosome 6q27, focusing on the genes in the region, to identify polymorphisms that may contribute to susceptibility to RA and potentially to other autoimmune diseases. Validated single nucleotide polymorphisms (SNPs; *n *= 65) were selected from public databases from the 330 kb region of *IDDM8*. These were genotyped using Sequenom MassArray genotyping technology in two datasets; the test dataset comprised 180 RA cases and 180 controls. We tested 50 SNPs for association with RA and any significant associations were genotyped in a second dataset of 174 RA cases and 192 controls, and the datasets were combined before analysis. Association analysis was performed by chi-square test implemented in Stata software and linkage disequilibrium and haplotype analysis was performed using Helix tree version 4.1. There was initial weak evidence of association, with RA, of a number of SNPs around the *loc154449 *putative gene and within the *KIAA1838 *gene; however, these associations were not significant in the combined dataset. Our study has failed to detect evidence of association with any of the known genes mapping to the *IDDM8 *locus with RA.

## Introduction

Rheumatoid arthritis (RA; MIM#180300) is a systemic autoimmune disease characterized by chronic inflammation of the joint synovium. In common with other autoimmune diseases, such as type 1 diabetes (T1D; MIM#222100), systemic lupus erythematosus (SLE; MIM#152700) and autoimmune thyroid disease, it is a complex disease caused by both genetic and environmental factors. Various lines of evidence suggest that some of the genetic factors may be common to a number of autoimmune diseases. These include their shared pathophysiology and also the co-occurrence of autoimmune diseases in families. In addition, observations from meta-analyses of autoimmune disease whole genome screens show non-random clustering of disease susceptibility loci for a number of human autoimmune diseases and animal models of autoimmunity [1,2]. Recently convincing proof of this hypothesis has been provided by the association of the missense single nucleotide polymorphism (SNP; rs2476601) in the protein tyrosine phosphatase N22 (*PTPN22*) gene with at least five autoimmune diseases; RA [3,4], SLE [5], autoimmune thyroid disease [6], T1D [7] and juvenile idiopathic arthritis [4].

**Figure 1:**
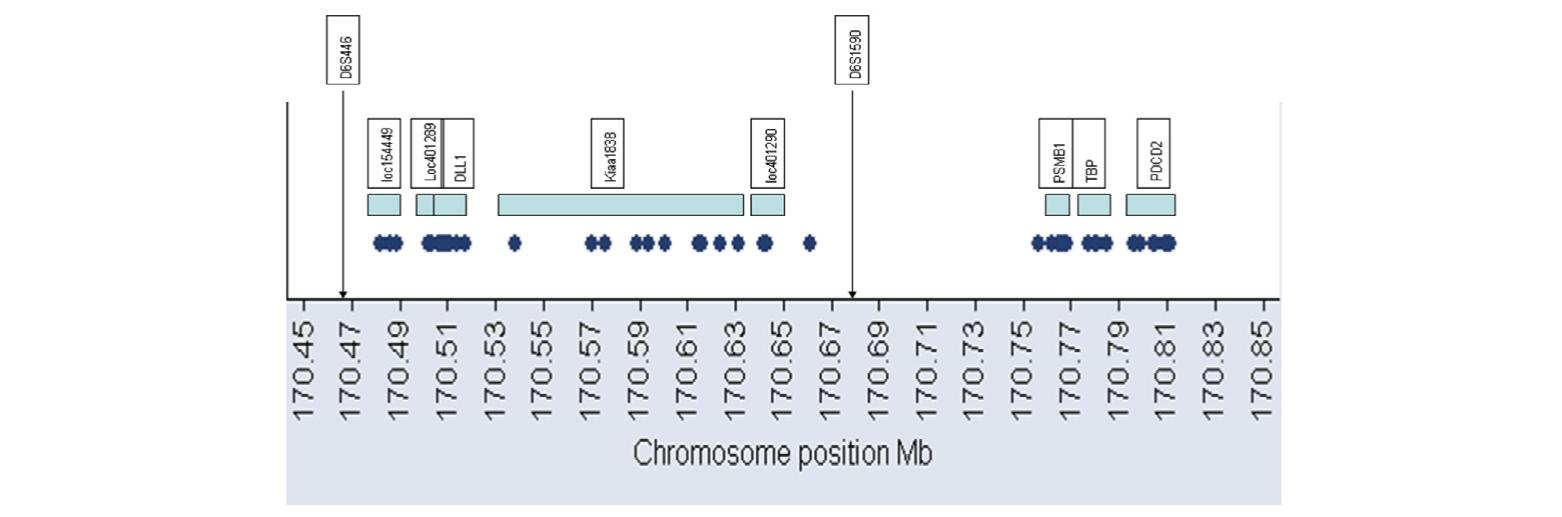
A schematic diagram of the *IDDM8 *region. The genes are shown in blue boxes, arrows denote position of the microsatellite markers associated in the Myerscough and colleagues study [9] and blue circles denote the single nucleotide polymorphisms.

We have, therefore, hypothesized that loci identified in one autoimmune disease are strong potential candidates in other related conditions. Of the autoimmune diseases that cluster within the same families as RA, T1D has been most thoroughly investigated for genetic susceptibility loci. The T1D susceptibility locus, denoted *IDDM8*, a region on chromosome 6q27 (Figure 1), spans approximately 200 kb and contains a number of potential candidate genes, including programmed cell death 2 (*PDCD2*), proteosome subunit beta type 1 (*PSMB1*), delta-like ligand 1 (*DLL-1*) and TATA box-binding protein (*TBP*) amongst others [8]. Interest in this region, in relation to RA, has stemmed from our previous work that revealed evidence for linkage and association of a microsatellite marker (D6S446) with RA in a dataset comprising RA affected sibling pair families and RA simplex families. An adjacent microsatellite, D6S1590, has also shown evidence of linkage and association with RA in the same families [9].

The aim of this present study was to fine map the *IDDM8 *region on chromosome 6q27. We have chosen to examine a 330 kb region spanning the *IDDM8 *region and have focused on the genes in this region to identify variants that may contribute to susceptibility to RA and potentially to other autoimmune diseases.

## Materials and methods

### Subjects

DNA was available for an initial RA dataset comprising 180 RA cases; these were combined with a further 174 RA cases to give a total RA dataset of 354 RA cases for the second stage analysis. The RA cases were obtained either from the ARC National Repository for families with RA or from clinics within the Greater Manchester area of Northern England. For patients obtained through the National Repository, only one affected case per family was selected at random for investigation. All RA cases had disease that satisfied the 1987 American college of Rheumatology criteria [10] modified for genetic studies [11]. Rheumatoid factor (RF) status was ascertained using a particle agglutination test, and a positive result was classified as a titre of 1 in 40 or greater. Of the RA cases used in this study, 75% were RF positive, 83% had erosive disease and the mean age-at-onset was 44.6 ± 14.6 years. *HLA-DRB1 *genotypes were determined using a commercially available semi-automated PCR-sequence specific oligonucleotide probe typing technique (INNO-LiPA; Abbott Laboratories, Maidenhead, UK). Of the RA cases, 16% had zero copies of the shared epitope, 47% had one copy and 34% had 2 copies (3% of cases not HLA typed).

The initial RA case cohort was compared with a cohort of 180 population control individuals; this was combined with a second cohort of 192 population control individuals to give a total control dataset of 372 controls for the second stage analysis. Population control subjects were recruited from blood donors and from General Practice registers.

All patients and controls were of UK Caucasoid ethnic origin, were recruited with ethical committee approval and provided informed consent.

### SNP selection

Over the 330 kb region of *IDDM8 *on chromosome 6q27, frequency validated SNPs were selected from public databases, including NCBI [12] and HapMap (CEPH population) [13], using a gene-focused approach. The genes in the region are *loc154449*, *loc401289*, *DLL1*, *KIAA1838*, *loc401290*, *PSMB1*, *TBP *and *PDCD2*. Information on linkage disequilibrium across the region was obtained from HapMap and, where genes fell within haplotype blocks, haplotype tagging SNPs were selected to reduce the total number of SNPs required for genotyping. All SNPs within coding regions or with any potential function were also prioritized for genotyping. In total, 65 SNPs were selected for genotyping. Polymorphisms were mapped to the UCSC genome browser [14] May 2004 human reference sequence based on NCBI build 35. Details of primer and probe sequences are available on request.

### Genotyping protocol

SNPs were genotyped using Sequenom MassArray genotyping technology, according to manufacturer's instructions, whereby the genomic sequence containing the SNP is amplified by PCR [15]. The amplified product is cleaned using shrimp alkaline phosphatase to neutralize any unincorporated dNTPs. This is followed by the homogeneous MassEXTEND process. This process utilizes a primer that anneals to the genomic amplification product immediately adjacent to the SNP site and is extended to generate SNP-specified DNA products of different length with predictable masses that can be resolved easily by mass spectrometry. Following the MassEXTEND reaction, SpectroCLEAN resin is added to the reaction mixture to remove extraneous salts that could interfere with MALDI-TOF mass spectrometry. The reaction mixture is then spotted onto a SpectroCHIP microarray and subjected to the MALDI-TOF mass spectrometry. SpectroTYPER software identifies the SNP-specific peaks according to their expected masses and automatically assigns the genotype calls.

### Statistical analysis

All SNPs were tested for Hardy-Weinberg equilibrium in cases and controls. Association of the *IDDM8 *SNPs was tested using the chi-squared test implemented in Stata (Stata, College Station, TX, USA).

Pairwise linkage disequilibrium (LD) measures of D' and LD correlation coefficient r^2 ^were calculated and plotted on a graph. Two- and three-marker haplotypic associations using a moving window approach were investigated using haplotype trend regression implemented in HelixTree™ version 4.1 (Golden Helix Inc., Bozeman, Montana, USA). Haplotypes were inferred using the expectation-maximization algorithm.

The Tagger option in the program Haploview 3.2 [16] was used to determine how many of the SNPs in the specified HapMap region had been successfully tagged by the SNPs that have been genotyped.

## Results

### Association analysis of *IDDM8 *SNPs

We excluded 15 SNPs from any subsequent analyses either because they were non-polymorphic in this study, had a call rate of <80% or as they showed deviation from Hardy-Weinberg equilibrium expectations in the control population (p < 0.001). For stage 1, 50 SNPs were analyzed for association with RA (Figure 1).

Following single marker analysis of the test dataset, one SNP mapping close to *loc154449 *showed a trend towards allelic association (rs11752069, p = 0.06) with RA and significant genotypic association with RA (p = 0.05). A second SNP mapping within the *KIAA1838 *gene was significantly associated with RA (rs910424, allelic association p = 0.012), whilst three other SNPs in *KIAA1838 *showed a trend towards association (p < 0.1) (Table 1).

Data from the HapMap suggest there is variable LD across the *IDDM8 *region. We therefore carried out two- and three-marker haplotype analysis for SNPs across the genes using HelixTree™ version 4.1 to see if evidence of association was stronger in two- or three-marker haplotypes. In the stage 1 dataset, analysis using Haplotype Trend Regression in Helix Tree™ showed evidence of association with RA of a number of two- and three-marker haplotypes within the *KIAA1838 *gene (Table 2). There was borderline significant association of a two-marker T_T haplotype (SNPs rs910425_rs910424), and a three-marker T_T_A haplotype (SNPs rs910425_rs910424_rs2881062) with RA (p values of 0.07 and 0.09, respectively). We therefore went on to genotype these SNPs, and other SNPs in the gene, in a second set of cases and controls. Two SNPs (rs2024694) and (rs958998) showed deviation from Hardy-Weinberg equilibrium in the combined controls and were not analyzed any further. These were combined with the data from stage 1 for the association analysis. In the analysis no SNPs or haplotypes were significant at a p value < 0.05 (Table 3 and 4).

### Evaluation of SNP coverage

**Figure 2:**
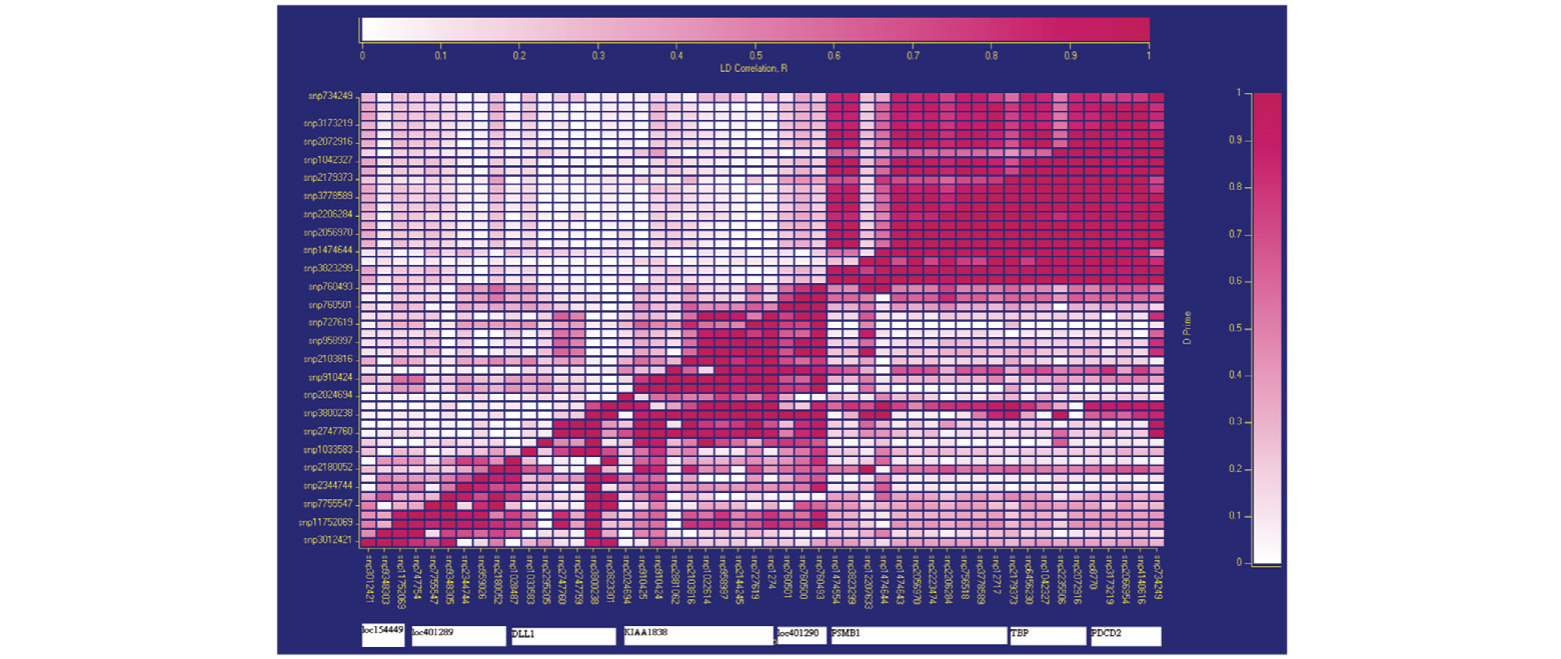
Linkage disequilibrium plot of the *IDDM8 *region in controls. The plot shows both linkage disequilibrium correlation and D' as measures of linkage disequilibrium across the *IDDM8 *region.

Calculations of pairwise LD between all markers examined were performed and plotted on a graph for the total region studied (Figure 2). A block of strong LD can be found at the distal end of the *IDDM8 *region, spanning SNPs rs1474554 to rs734249 and approximately 55 kb. The genes *PSMB*, *TBP *and *PDCD2 *map within this block.

Figure 1 shows a schematic diagram of the region studied with the 50 intragenic SNPs plotted across the chromosomal region.

Results from Tagger (implemented in Haploview version 3.2) suggest that, for the *PSMB1-TBP-PDCD2 *gene region, genotyping the 11 SNPs within this region captured all of the 21 HapMap SNPs with r^2 ^> 0.8. For the *KIAA1838 *gene the 10 SNPs genotyped in this study captured 33 of the 38 HapMap SNPs with r^2 ^> 0.8. For the *loc401290 *gene region the 3 SNPs captured all of the 9 HapMap SNPs with r^2 ^> 0.8. There is, therefore, good coverage of the *PSMB1-TBP-PDCD2 *gene region, *KIAA1838 *and *LOC401290*. Variation across two other gene regions was less well captured by the SNPs we analyzed. The *loc154449 *gene region falls outside LD blocks so 3 SNPs were selected to span the gene and are located, on average, 6.8 kb apart. The *DLL1 *gene also falls outside a haplotype block; therefore, SNPs spanning the gene were selected. The seven SNPs that were used in the analysis spanning the *DLL1 *gene had an average spacing of 1.4 kb.

## Discussion

Linkage to the *IDDM8 *region on chromosome 6q27 was originally identified in the first whole genome screen in T1D [17] and the region has also been linked to multiple sclerosis [18] and SLE [19], supporting the hypothesis that it could harbor polymorphisms important in autoimmunity. Linkage disequilibrium mapping of the region in T1D narrowed down the region to the terminal 200 kb of chromosome 6q spanning the *PDCD2*-*TBP*-*PSMB1 *gene complex [8]. Previous investigation of the region in RA found evidence of linkage and association to two microsatellite markers (D6S446 and D6S1590) [9].

In this study we have taken a SNP-based association mapping approach and selected a large number of SNPs spanning the known genes in this region. Despite initial weak evidence of association with RA of a number of SNPs around the *loc154449 *putative gene and within the *KIAA1838 *gene, there was no evidence of association with RA in the combined dataset and we conclude that there is no evidence to support association of polymorphisms in these genes with RA.

Information on LD across the region was obtained from the HapMap. Results from the software program Tagger suggest that the SNPs selected within the *PDCD2*-*TBP*-*PSMB1 *gene region and the *loc401289 *gene region capture all the HapMap SNPs within these regions, suggesting that adequate coverage of the region was achieved with the SNPs analyzed in this study.

The *PDCD2*-*TBP*-*PSMB1 *gene region was initially highlighted in the T1D study as likely to contain the susceptibility gene [8]. A recent study of the *IDDM8 *region in T1D, however, found no evidence of association, although they could not completely rule out the possibility that the putative *IDDM8 *locus exists elsewhere in this chromosomal region [20]. Other genes in the *IDDM8 *region include the *KIAA1838 *gene and, although the 10 SNPs within this gene captured 33 out of 38 SNPs identified on the HapMap, there is a possibility that variation across the region has not been completely captured and further SNPs would need to be genotyped before this locus can be confidently excluded for modest effect sizes.

Another possible reason for the failure to identify a susceptibility region in the study could be heterogeneity between the dataset used in this study and the dataset used in the previous analysis [9]. However, half of the cases used in this study were probands from the National Repository of RA cases that had been used in the previous study and there were no differences in gender (p = 0.06) or severity of disease (as denoted by number of erosions; p = 0.55). However, there was a significant difference in RF status between the two subgroups (p = 0.03); of the RA probands 86% had RF whilst the dataset used in this study had 76%.

The total dataset analyzed in this study (354 cases and 372 controls) had the power to detect an effect size or odds ratio greater than 1.6; therefore, if the *IDDM8 *region conferred a risk similar to that of *PTPN22 *in RA (odds ratio = 1.8), then we would have had 80% power to detect it (p = 0.05). However, for smaller effect sizes, such as that of *CTLA4 *in T1D (odds ratio = 1.14) then our study would have been underpowered.

Our study has failed to detect evidence of association with any of the known genes mapping to the *IDDM8 *locus, a region we had identified as a candidate autoimmune locus common to RA, T1D and SLE. It is possible that the limits of the region defined by earlier T1D studies have, in fact, failed to encompass the RA susceptibility gene that gave rise to evidence of linkage and association to microsatellite markers in our initial study, and future studies would need to focus on genes adjacent to those investigated here.

## Conclusion

Our study has failed to detect evidence of association with any of the known genes mapping to the *IDDM8 *locus with RA.

## Abbreviations

DLL-1 = Delta-like ligand 1; LD = linkage disequilibrium; PDCD2 = programmed cell death 2; PSMB1 = proteosome subunit beta type 1; PTPN22 = protein tyrosine phosphatase N22; RA = rheumatoid arthritis; RF = rheumatoid factor; SLE = systemic lupus erythematosus; SNP = single nucleotide polymorphism; T1D = type 1 diabetes; TBP = TATA-box binding protein.

## Competing interests

The authors declare that they have no competing interests.

## Authors' contributions

AH planned the work, carried out all the laboratory work, the statistical analysis and also helped draft the manuscript. AB participated in the study design and helped to draft the manuscript. SJ participated in the study design and statistical analysis. NS participated in statistical analysis. JW participitated in the study design and helped draft the manuscript. All authors read and approved the final manuscript.

## Figures and Tables

**Table 1:** *IDDM8 *single nucleotide polymorphism allele frequencies in 180 rheumatoid arthritis cases and 180 controls

SNP	Gene	Allele frequencies in RA cases	Allele frequencies in controls	OR (95 percent CI)	P value	Genotype frequencies in RA cases	Genotype frequencies in controls	P value^a^
rs11752069	*LOC154449*	C = 0.35 (112) G = 0.65 (204)	C = 0.29 (94) G = 0.71 (234)	1.37 (0.98–1.9)	0.065	CC = 0.11 (17) CG = 0.49 (78) GG = 0.4 (63)	CC = 0.1 (17) CG = 0.37 (60) GG = 0.53 (87)	0.05
rs910424	*KIAA1838*	C = 0.63 (203) T = 0.37 (119)	C = 0.72 (248) T = 0.28 (96)	1.51 (1.1–2.1)	0.012	CC = 0.39 (63) CT = 0.48 (77) TT = 0.13 (21)	CC = 0.52 (90) CT = 0.4 (68) TT = 0.08 (14)	0.04
rs958997	*KIAA1838*	A = 0.9 (289) C = 0.1 (31)	A = 0.86 (290) C = 0.14 (48)	1.54 (0.96–2.5)	0.075	AA = 0.82 (131) AC = 0.17 (27) CC = 0.01 (2)	AA = 0.73 (124) AC = 0.25 (42) CC = 0.02 (3)	0.18
rs2144245	*KIAA1838*	C = 0.87 (245) T = 0.13 (35)	C = 0.82 (252) T = 0.18 (54)	1.5 (0.95–2.4)	0.083	CC = 0.76 (106) CT = 0.24 (33) TT = 0.007 (4)	CC = 0.67 (103) CT = 0.3 (46) TT = 0.03 (4)	0.18
rs1274	*KIAA1838*	A = 0.9 (291) G = 0.1 (31)	A = 0.86 (299) G = 0.14 (49)	1.54 (0.96–2.5)	0.076	AA = 0.82 (132) AG = 0.17 (27) GG = 0.01 (2)	AA = 0.73 (128) AG = 0.25 (43) GG = 0.02 (3)	0.18

^a^P value calculated from chi-squared comparison of genotype frequencies in case versus controls. CI, confidence interval; OR, odds ratio; RA, rheumatoid arthritis; SNP, single nucleotide polymorphism.

**Table 2:** *KIAA1838 *two- and three-marker haplotype analysis in 180 rheumatoid arthritis cases and 180 controls

	Associated haplotype	Haplotype frequency in cases (percentage)	Haplotype frequency in controls (percentage)	Haplotype chi-square (P value^a^)
Two-marker haplotype				
rs910425_rs910424	T_T	36.4	28.0	3.26 (p = 0.07)
rs2024694_rs910425	G_T	40.9	34.1	2.0 (p = 0.16)
Three-marker haplotype				
rs2024694_rs910425_rs910424	G_T_T	32.7	25.6	2.27 (p = 0.13)
rs910425_rs910424_rs2881062	T_T_A	36.4	28.1	2.85 (p = 0.09)

^a^P value calculated from chi-squared comparison of haplotype frequencies in case versus controls.

**Table 3:** *IDDM8 *single nucleotide polymorphism allele frequencies in 354 rheumatoid arthritis cases and 372 controls

SNP	Gene	Allele frequencies in RA cases	Allele frequencies in controls	OR (95 percent CI)	P value	Genotype frequencies in RA cases	Genotype frequencies in controls	P value^a^
rs11752069	*LOC154449*	C = 0.32 (190) G = 0.68 (404)	C = 0.31 (215) G = 0.69 (479)	1.07 (0.84–1.4)	0.57	CC = 0.09 (28) CG = 0.45 (134) GG = 0.46 (135)	CC = 0.1 (36) CG = 0.40 (140) GG = 0.49 (171)	0.47
rs910424	*KIAA1838*	C = 0.67 (419) T = 0.33 (207)	C = 0.70 (493) T = 0.30 (211)	1.13 (0.89–1.4)	0.3	CC = 0.44 (139) CT = 0.45 (141) TT = 0.11 (33)	CC = 0.49 (173) CT = 0.41 (144) TT = 0.10 (35)	0.47
rs958997	*KIAA1838*	A = 0.90 (553) C = 0.10 (61)	A = 0.87 (606) C = 0.13 (90)	1.29 (0.9–1.86)	0.13	AA = 0.81 (249) AC = 0.17 (53) CC = 0.02 (5)	AA = 0.76 (263) AC = 0.23 (80) CC = 0.01 (5)	0.19
rs2144245	*KIAA1838*	C = 0.88 (514) T = 0.12 (70)	C = 0.86 (585) T = 0.14 (95)	1.24 (0.87–1.7)	0.21	CC = 0.78 (229) CT = 0.20 (59) TT = 0.02 (4)	CC = 0.74 (252) CT = 0.24 (82) TT = 0.02 (6)	0.18
rs1274^a^	*KIAA1838*	A = 0.9 (291) G = 0.1 (31)	A = 0.86 (299) G = 0.14 (49)	1.54 (0.96–2.5)	0.076	AA = 0.82 (132) AG = 0.17 (27) GG = 0.01 (2)	AA = 0.73 (128) AG = 0.25 (43) GG = 0.02 (3)	0.18

^a^P value calculated from chi-squared comparison of genotype frequencies in case versus controls. CI, confidence interval; OR, odds ratio; RA, rheumatoid arthritis; SNP, single nucleotide polymorphism.

**Table 4:** *KIAA1838 *two- and three-marker haplotype analysis in 354 rheumatoid arthritis cases and 372 controls

	Associated haplotype	Haplotype frequency in cases (percentage)	Haplotype frequency in controls (percentage)	Haplotype chi-square (P value^a^)
Two-marker haplotype				
rs910425_rs910424	T_T	32.8	30.5	0.48 (p = 0.48)
Three-marker haplotype				
rs910425_rs910424_rs2881062	T_T_A	32.8	30.6	0.38 (p = 0.54)

^a^P value calculated from chi-squared comparison of haplotype frequencies in case versus controls.

## References

[B1] Vyse TJ, Todd JA (1996). Genetic analysis of autoimmune disease. Cell.

[B2] Becker KG, Simon RM, Bailey-Wilson JE, Freidlin B, Biddison WE, McFarland HF, Trent JM (1998). Clustering of non-major histocompatibility complex susceptibility candidate loci in human autoimmune diseases. Proc Natl Acad Sci USA.

[B3] Begovich AB, Carlton VE, Honigberg LA, Schrodi SJ, Chokkalingam AP, Alexander HC, Ardlie KG, Huang Q, Smith AM, Spoerke JM (2004). A missense single-nucleotide polymorphism in a gene encoding a protein tyrosine phosphatase (PTPN22) is associated with rheumatoid arthritis. Am J Hum Genet.

[B4] Hinks A, Barton A, John S, Bruce I, Hawkins C, Griffiths CE, Donn R, Thomson W, Silman A, Worthington J (2005). Association between the PTPN22 gene and rheumatoid arthritis and juvenile idiopathic arthritis in a UK population: Further support that PTPN22 is an autoimmunity gene. Arthritis Rheum.

[B5] Kyogoku C, Langefeld CD, Ortmann WA, Lee A, Selby S, Carlton VE, Chang M, Ramos P, Baechler EC, Batliwalla FM (2004). Genetic association of the R620W polymorphism of protein tyrosine phosphatase PTPN22 with human SLE. Am J Hum Genet.

[B6] Velaga MR, Wilson V, Jennings CE, Owen CJ, Herington S, Donaldson PT, Ball SG, James RA, Quinton R, Perros P (2004). The codon 620 tryptophan allele of the lymphoid tyrosine phosphatase (LYP) gene is a major determinant of Graves' disease. J Clin Endocrinol Metab.

[B7] Bottini N, Musumeci L, Alonso A, Rahmouni S, Nika K, Rostamkhani M, MacMurray J, Meloni GF, Lucarelli P, Pellecchia M (2004). A functional variant of lymphoid tyrosine phosphatase is associated with type I diabetes. Nat Genet.

[B8] Owerbach D (2000). Physical and genetic mapping of IDDM8 on chromosome 6q27. Diabetes.

[B9] Myerscough A, John S, Barrett JH, Ollier WE, Worthington J (2000). Linkage of rheumatoid arthritis to insulin-dependent diabetes mellitus loci: evidence supporting a hypothesis for the existence of common autoimmune susceptibility loci. Arthritis Rheum.

[B10] Arnett FC, Edworthy SM, Bloch DA, McShane DJ, Fries JF, Cooper NS, Healey LA, Kaplan SR, Liang MH, Luthra HS (1988). The American Rheumatism Association 1987 revised criteria for the classification of rheumatoid arthritis. Arthritis Rheum.

[B11] MacGregor AJ, Bamber S, Silman AJ (1994). A comparison of the performance of different methods of disease classification for rheumatoid arthritis. Results of an analysis from a nationwide twin study. J Rheumatol.

[B12] The National Centre for Biotechnology Information. http://www.ncbi.nlm.nih.gov/.

[B13] The HapMap. http://www.hapmap.org/.

[B14] UCSC Genome Bioinformatics site. http://genome.cse.ucsc.edu/.

[B15] Sequenom. http://www.sequenom.com/applications/high_performance_genotyping.php.

[B16] Haploview. http://www.broad.mit.edu/mpg/haploview/.

[B17] Davies JL, Kawaguchi Y, Bennett ST, Copeman JB, Cordell HJ, Pritchard LE, Reed PW, Gough SCL, Jenkins SC, Palmer SM (1994). A genome-wide search for human type-1 diabetes susceptibility genes. Nature.

[B18] Haines JL, Ter Minassian M, Bazyk A, Gusella JF, Kim DJ, Terwedow H, Pericak-Vance MA, Rimmler JB, Haynes CS, Roses AD (1996). A complete genomic screen for multiple sclerosis underscores a role for the major histocompatability complex. The Multiple Sclerosis Genetics Group. Nat Genet.

[B19] Moser KL, Neas BR, Salmon JE, Yu H, Gray-McGuire C, Asundi N, Bruner GR, Fox J, Kelly J, Henshall S (1998). Genome scan of human systemic lupus erythematosus: evidence for linkage on chromosome 1q in African-American pedigrees. Proc Natl Acad Sci USA.

[B20] Payne F, Smyth DJ, Pask R, Cooper JD, Masters J, Wang WY, Godfrey LM, Bowden G, Szeszko J, Smink LJ (2005). No evidence for association of the TATA-box binding protein glutamine repeat sequence or the flanking chromosome 6q27 region with type 1 diabetes. Biochem Biophys Res Commun.

